# Village Building Identification Based on Ensemble Convolutional Neural Networks

**DOI:** 10.3390/s17112487

**Published:** 2017-10-30

**Authors:** Zhiling Guo, Qi Chen, Guangming Wu, Yongwei Xu, Ryosuke Shibasaki, Xiaowei Shao

**Affiliations:** 1 Center for Spatial Information Science, University of Tokyo, Kashiwa 277-8568, Japan; guozhilingcc@csis.u-tokyo.ac.jp (Z.G.); qichen@csis.u-tokyo.ac.jp (Q.C.); huster-wgm@csis.u-tokyo.ac.jp (G.W.); xyw@csis.u-tokyo.ac.jp (Y.X.); shiba@csis.u-tokyo.ac.jp (R.S.); 2 Faculty of Information Engineering, China University of Geosciences (Wuhan), Wuhan 430074, China

**Keywords:** Ensemble Convolutional Neural Networks, remote sensing, building detection, village mapping, multiscale feature learning

## Abstract

In this study, we present the Ensemble Convolutional Neural Network (ECNN), an elaborate CNN frame formulated based on ensembling state-of-the-art CNN models, to identify village buildings from open high-resolution remote sensing (HRRS) images. First, to optimize and mine the capability of CNN for village mapping and to ensure compatibility with our classification targets, a few state-of-the-art models were carefully optimized and enhanced based on a series of rigorous analyses and evaluations. Second, rather than directly implementing building identification by using these models, we exploited most of their advantages by ensembling their feature extractor parts into a stronger model called ECNN based on the multiscale feature learning method. Finally, the generated ECNN was applied to a pixel-level classification frame to implement object identification. The proposed method can serve as a viable tool for village building identification with high accuracy and efficiency. The experimental results obtained from the test area in Savannakhet province, Laos, prove that the proposed ECNN model significantly outperforms existing methods, improving overall accuracy from 96.64% to 99.26%, and kappa from 0.57 to 0.86.

## 1. Introduction

Given that accurate building maps are often unavailable or are outdated in undeveloped village areas, building identification in such areas has become a significant research field in remote sensing [1]. Insufficient building information in village leads to inconvenience and has several negative consequences [2]. First, in the event of a catastrophe, building maps are indispensable [3]. For instance, during catastrophic events such as the aftermath of the 2011 Tōhoku earthquake and tsunami [4], land conditions change rapidly with secondary disasters such as landslides, tsunamis, and continual aftershocks [5]. To save victims and provide disaster relief in a convenient way, it is important to swiftly update the locations of residential buildings and information about other land features. Furthermore, in village planning, which aims to benefit village inhabitants, public facilities need to be developed based on information about the distribution of residential buildings [6]. In this study, we define village buildings as any settlement spread out over a length of 2 km. In contrast to densely packed urban buildings, village buildings have distinct characteristics, for instance, they are sparsely scattered, change arbitrarily owing to the lack of regulation, and do not have distinct architectural features. Moreover, village buildings are usually mixed with complex and diverse land features such as agricultural lands, mountains, and rivers [7]. Such complexity of spatial and structural patterns makes village building identification a fairly challenging problem, and the usage of building maps ensures that the tools used for building identification provide rapid, accurate, efficient, and time-sequenced results.

With the rapid development of remote sensing satellite imaging techniques in recent years, a considerable number of highly spatially resolved images are available [8,9,10]. Owing to the high price–performance ratio, many remote sensing image classification studies are performed using open high-resolution remote sensing (HRRS) data [11,12,13]. In this study, three-band HRRS images from Google Earth (GE) [14] and Bing Maps [15] are used as the data source and applied to village building mapping in a large rural region.

Recently, deep convolutional neural networks (CNN) have been successfully applied to many pattern-recognition tasks [16]. Compared with most existing classification methods, which can only generate low- or middle-level image features with limited representation ability, CNN does not require prior manual feature extraction [17,18]. A large volume of abstract features can be extracted automatically based on gradient descent and back propagation algorithms, thus resulting in higher accuracy and efficiency [19,20].

CNN-based pixel-level classification is one of the most important and popular topics in the geoscience and remote sensing community, and it can be used to efficiently identify individual land features in greater detail, and significant progress to this end has been achieved in recent years [21,22,23]. Related works have been introduced in our previous work [24]. In this study, we focus on the use of CNN for pixel-level [25] classification via HRRS images according to our previous work, according to which, pixel-level village building identification is implemented based on a shallow CNN structure that can achieve relatively high accuracy when using GE images compared to other machine learning methods. Although the previous CNN structure proved to be very useful for exploring features and classification, the unstable performance in some study areas indicates that it might be inadequate for exploiting the full potential capability of CNN.

Identification performance depends highly on the structure of the CNN model [26]. To adapt CNN for village building identification with high accuracy, we can apply state-of-the-art models such as AlexNet [27], VGGNet [28], GoogLeNet [29], SqueezeNet [30] achieved in ImageNet [31] Large-Scale Visual Recognition Challenge (ILSVRC) [26]. The high feasibility of applying the aforementioned models has been proven by many studies in different fields [32,33,34,35,36]. To make the most of these mentioned state-of-the-art models and to ensure compatibility with our experiment, we optimized and enhanced their architectures into four self-designed structures named AlexNet-like, VGGNet-like, GoogLeNet-like and SqueezeNet-like via rigorous experiment while fully considering the characteristics of the input HRRS images and identification targets. The identification capability of an individual optimized CNN model is limited. To make the most of the single feature extraction capability, a promising solution would be to create an ensemble of several CNN models. In this study, we employ multiscale feature learning [37] to achieve the goal.

Multiscale feature learning schemes such as recurrent neural networks (RNNs) [38] and scene parsing using CNNs [39] have been showing tremendous capabilities in different tasks. In multiscale feature learning, several paralleled CNN models of varying contextual input size are implemented to extract features, and thereafter, the output of each CNN is ensembled and concatenated into a classifier. In practice, Martin et al. [40] implemented multi-class land feature classification by using four stacked CNN models. To improve and smooth semantic image segmentation, Marmanis et al. [41] and Farabet et al. [42] implemented multi-scale segmentation-based parallel CNN architectures. Richard et al. [43] and Pedro et al. [44] achieved multiscale feature learning by stacking multiple shallow networks with tied convolution weights on top of each other. Ding et al. [45] combined deep CNN with multiscale feature for intelligent spindle bearing fault diagnosis. In the case of medical image processing, Kiros et al. [46] utilized stacked multiscale feature learning for massive feature extraction and Tom et al. [47] for a deep 3D convolutional encoder. Many studies have used RGB-D images to implement classification and segmentation, [48,49,50,51], rather than inputting a four-dimensional image into a single CNN in a directed way; features of information in RGB and depth bands are usually extracted based on well designed parallel CNNs respectively. Finally, the obtained features are merged in a fully-connected layer for implementing different tasks. The multiscale feature learning method can be used effectively not only in the computer vision field but also in other fields such as recommender systems [52], in which different features of data types such as text, image, social relationship, and user information are extracted using parallel CNNs; the final recommendation is provided based on classification of the ensembled features [53,54,55].

In this study, we present an elaborate formulated CNN model called Ensemble Convolutional Neural Networks(ECNN). Different from related works, ECNN achieves multiscale feature learning by ensembling the feature extractor part of four optimized state-of-the-art models, and we apply it to implement the pixel-level village building identification task.

The main contributions of this study can be summarized as follows:

- We explored how to construct CNN architecture that can adapt to the village building identification task based on insightful and in-depth analysis.
- We optimized state-of-the-art CNN models by using rigorous principles to explore their potential for pixel-level building identification via HRRS images.
- We presented a novel CNN frame called ECNN based on multiscale feature learning by emsembling parallel optimized state-of-the-art CNN models.
- We implemented the proposed method for village building identification and found that it outperforms the existing state-of-the-art methods, achieving an overall accuracy and kappa coefficient of 99.26% and 0.86 respectively.

The remainder of this paper is organized as follows. In Section 2, we describe the study area and the experimental dataset. Details about the methods are presented in Section 3. In Section 4, we present the experimental results and discuss the capability of the proposed method in comparison to existing methods. Finally, we present our conclusions and a few proposals for future work in Section 5.

## 2. Data Source

### 2.1. Study Area

**Figure 1:**
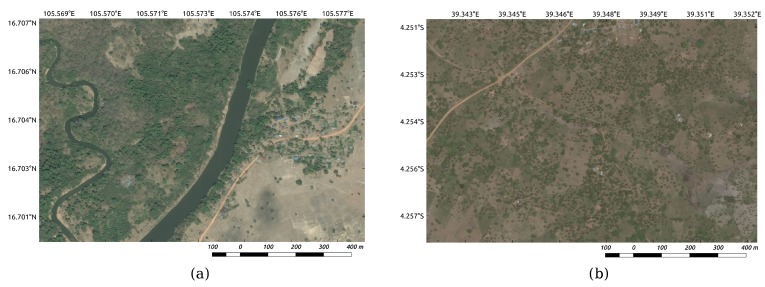
Study area example (**a**), located in Savannakhet Province, Laos, shows abundant land features; Study area example (**b**), located in Kwale Province, Kenya, with relatively desolate land features. The resolution of all images is 1.2 m.

To test the feasibility of the proposed method in different regions and by using different data sources, we selected rural areas in developing countries such as Laos and Kenya. One of the study areas is located in Kaysone, Savannakhet province in Laos. Its longitude and latitude range from E$104^{\circ }47^{\prime }22\prime \prime$ to E$104^{\circ }49^{\prime }54\prime \prime$ and from N$16^{\circ }34^{\prime }28\prime \prime$ to N$16^{\circ }36^{\prime }26\prime \prime$, respectively, and it measures approximately 12.08 km${}^{2}$. The study area was a complex rural region with many different types of landscape, including abundant natural components such as mountains, rivers, and vegetation cover, as well as artificial areas such as villages, roads, and cultivated land, which are typical of rural areas. The other study area was Kwale, a small town in the capital of Kwale County, Kenya. It is located at around S$4^{\circ }10^{\prime }28\prime \prime$ and E$39^{\circ }27^{\prime }37\prime \prime$, 30 km southwest of Mombasa and 15 km inland, and it measures approximately 30.20 km${}^{2}$. The area was mainly covered by forest and other desolate landscapes, and the buildings were rather scattered. A few samples from the study area are shown in Figure 1.

### 2.2. Data

The remote top-view RGB image of Kaysone and Kwale, both with a resolution of 1.2 m, were captured from Google’s satellite map in February 2016 and Bing Maps in January 2016, respectively. As the training dataset for Laos, we deliberately selected a few typical village/non-village areas from the data source. In village areas, the training dataset mainly showed land features such as buildings, roads, rivers, and cultivated lands, while in non-village areas, mountains, forests, and vegetation cover are the main features. The ground truth map of the village buildings was manually drawn beforehand by using a polygon-based interaction tool. This ground truth map contained accurate information of the land categories and was chiefly used for sampling and result detection. Similar to Laos, the training dataset in Kenya was also selected considering the characteristics and the diversity of the landscape. The test dataset contained the entire testing area of Laos and Kenya, and several different types of landscape were shown; the land features in different countries and areas showed distinctive characteristics. As shown in Figure 1, land features in Laos (Figure 1a) are more abundant than those in Kenya (Figure 1b). The diversity and complexity of the images also makes the identification task difficult. This, in turn, warrants that the classification model incorporate all these conditions.

## 3. Methods

**Figure 2:**
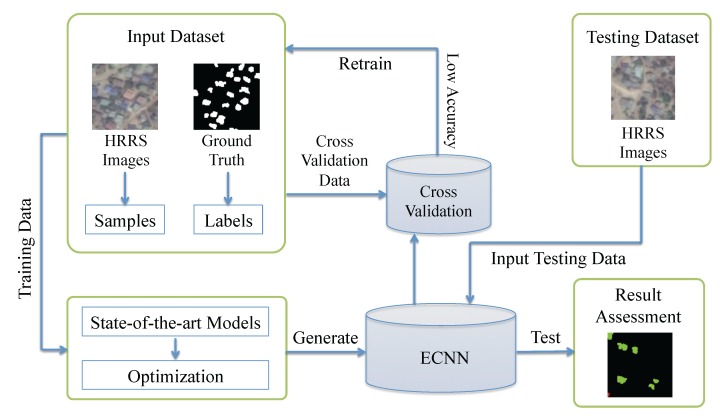
Workflow.

Figure 2 shows details of the workflow employed in our experiment. First, as introduced in Section 2.2, the training dataset in our experiment contains two parts: three-band RGB HRRS images and the corresponding ground truth labels. Importantly, both the complexity and characteristics of the identification target, and the diversity of land features need to be considered when preparing the dataset [56]. Second, to optimize and mine the capability of CNN for rural environmental building identification and ensure compatibility with our classification targets, a few state-of-the-art CNN structures were carefully optimized and enhanced based on a series of rigorous testing results. Then, we generated the ECNN model from the ensembling based on the identification capability of the CNN models. Third, depending on the back propagation and the gradient descent algorithms, the proposed ECNN structure can learn from the training dataset patterns that map the variables to the target and output a trained ECNN model that captures these relationships and can identify buildings in rural environments. Thereafter, cross validation [57] was implemented to verify the feasibility and performance of the CNN models; here, to evaluate the accuracy and reliability of the result, we used the confusion matrix [58], kappa coefficient [59] and overall accuracy. Finally, the generated ECNN model was applied to the prepared testing HRRS dataset to identify village buildings.

### 3.1. Convolutional Neural Networks

The CNN method is more robust and yields better performance than other machine learning methods in image pattern recognition owing to its capability in mining deep representative information from low-level inputs [28]. A single CNN model performs the steps of convolution [60], non-linear activation [61], and pooling [62]. With multilayer networks trained by gradient descent and back propagation algorithms, CNN can learn complex and nonlinear mapping from a high- to low-dimensional feature space [63].

In this experiment, the input dataset $x\in R^{h\times w\times c}$ refers to multichannel HRRS images, where each dimension represents the height, width, and number of channels. The output classification result $y\in R^{h^{\prime }\times w^{\prime }\times c^{\prime }}$ generated by y=H(x,Θ), where Θ denotes a set of parameters called kernels.

In the convolution layer, the input *x* with bias $\alpha \in R^{c^{\prime }}$ is computed by convolutional kernels $\Theta \in R^{\tilde{h}\times \tilde{w}\times \tilde{c}\times c^{\prime }}$. This computation can be formulated as follows:

(1) $$y_{i^{\prime }j^{\prime }k^{\prime }}=H\left(\alpha_{k^{\prime }}+\sum_{i=1}^{\tilde{h}}\sum_{j=1}^{\tilde{w}}\sum_{d=1}^{c}\Theta_{ijdk^{\prime }}\times x_{i^{\prime }+i,j^{\prime }+j,d}\right)$$

where H(·) denotes a nonlinear function to generate the hypothesis; instead of saturated activation methods, here, we use the rectified linear unit (ReLU):

(2) $$y_{ijk}=max\left\{0,x_{ijk}\right\}$$

To implement the subsampling operation, the max-pooling layer [64], which computes the maximum response of each image channel in a $\tilde{h}\times \tilde{w}$ subwindow, is used, and it is calculated as follows:

(3) $$y_{i^{\prime }j^{\prime }k}=\max_{1<i<\tilde{h},1<j<\tilde{w}}x_{i^{\prime }+i,j^{\prime }+j,k}$$

Finally, the classification result can be generated using the softmax function [65]:

(4) $$y_{ijk}=\frac{exp(x_{i,j,k})}{\sum_{d=1}^{c}exp\left(x_{ijd}\right)}$$

### 3.2. Model Optimization

In our previous study [24], the identification task was implemented using a simple CNN structure, in which, the sample window size was 18 × 18, and two convolutional layers followed by average pooling were implemented with 6 and 12 filters, respectively. Compared with other machine learning methods, although the preceding CNN structure is very feasible for the purposes of feature exploration and classification, it might not be effective for mining the complete capability of CNN. In this section, we aim to optimize the CNN model to achieve better results.

ILSVRC is an annual competition held by ImageNet since 2010, in which research teams submit programs that classify and detect objects and scenes. It is important to note that in 2012, AlexNet reduced the error rate to 16% from the previous best of 25%, and in the next couple of years, more accurate pattern recognition results were obtained using popular models such as GoogLeNet, VGGNet, SqueezeNet and ResNet [66].

To make the most of these aforementioned state-of-the-art models, we optimized their architectures by considering the characteristics of the input HRRS images and our identification targets. Here, we propose self-designed structures called AlexNet-like, GoogLeNet-like, VGGNet-like and SqueezeNet-like based on rigorous experiments and theories; thereafter, we ensemble these CNN models into ECNN.

The principle of optimizing CNN architecture is highly based on analyzing the learning curves of both the training and the cross validation results [67]. In addition to accuracy, two other important indexes need to be pointed out: bias and variance [68].

In this experiment, both bias and variance lead to severe problems. High bias can cause an algorithm to miss the relevant relationships between features and target outputs. Here are some ways to solve this challenge:

- Optimize the accuracy of the input training data. This means the training HRRS images and the corresponding labels of buildings and other land features must be as accurate as possible.
- Decrease the regularization coefficient λ [69], because doing so can solve under-fitting-related problems.
- Add number of features, such as implementation of higher-level CNN structures, which could extract more features

When facing high variance, which leads to over-fitting [70], the problem can be solved by:

- Adding more training samples would be helpful. Data augmentation such as adding more training HRRS images to the dataset considering the diversity.
- Increase the regularization coefficient λ, which can solve over-fitting problems.
- Decrease the number of features, by using a method such as Dropout [71].

Here, we optimize our model based on the preceding principles. We take the VGGNet-like (introduced in Section 3.4) structure as an example to explore how to configure the CNN architecture based on the characteristics of VGGNet. The final promising structure is generated by gradually enhancing and optimizing a simple initial CNN. Considering the experimental requirement, the three parameters to be evaluated in our experiment are number of filters, depth of architecture, and input sample window size. These parameters are connected in a way that determines the total number of units and the weight values of the entire structure.

The initial architecture is based on the basic CNN model utilized in our previous work. To enhance the architecture, the number of filters is configured by multiplying the original number of filters by *f* = [3 9 25 100 200]. The number of added convolutional layers is donated by *y*, and it ranges from 2 to 12 in steps of 2; the window size *s* is the area surrounding the pixel to be classified and is set to be between 14 and 50 with an interval of 2. We evaluated the effects of each parameter in terms of accuracy, efficiency, and learning curve; then, we integrated all the optimal settings to obtain a promising VGGNet-like architecture.

#### 3.2.1. Influence of Filter

**Figure 3:**
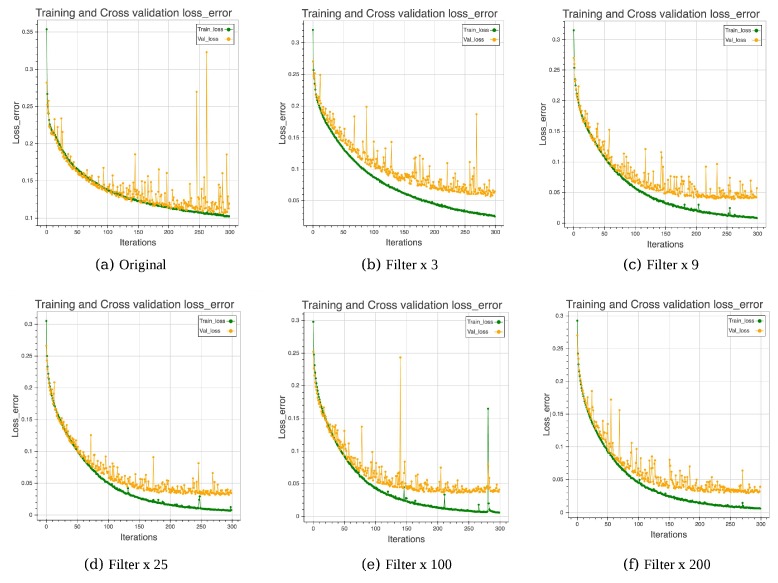
Influence of number of filters.

[-15]In general, the greater the number of filters, the greater the number of features that can be extracted. Here, we gradually increase the number of features from the original to *f* = [3, 9, 25, 100, 200] times in each convolutional layer. As shown in Table 1, when the number of filters reaches 25 times, the best training and testing results can be generated, and the model can achieve 98.98% and 0.83 in terms of testing accuracy and kappa value, respectively. Moreover, from the learning curve (Figure 3), until 200 and 300 times, the model does not encounter the challenge of over-fitting, which means that the number of features has not saturated yet. Upon adding more filters, the model tends to converge faster. However, when considering both accuracy and efficiency, the number of filters that can obtain a good enough result would be suitable.

Although high accuracy could be achieved, the model continued to suffer from unstable convergence, and it was not stable even after adding 200 times the original number of filters. The influence of depth of architecture will be explored in the next section.

#### 3.2.2. Influence of Depth

CNNs constitute a very important branch of deep learning. The preponderance of CNNs is highly based on the depth of architecture. By mining deeper and more abstract features and information from an identification target, usually, a very deep network can achieve higher accuracy. In recent years, owing to the improvement in the computational capability and hardware, it has become possible to construct and compute very deep networks. Recent state-of-the art architectures such as VGGNet and ResNet make the most of this principle.

In this experiment, to explore the effect of depth on the CNN model for identification of buildings in rural environments, we increased the number of convolutional layers from the original 2 to 14 in steps of 2. Both the training testing settings and the results are shown in Table 2.

At the outset, model accuracy increases as the network depth increases. However, when the number of convolutional layers is higher than 12, the network becomes stocked and even loses its identification capability. After rigorous analysis, we found that this problem is caused by gradient vanishing [72]. As we know, CNN is based on gradient descent and back propagation. When implementing the gradient descent algorithm, the input signal will be activated by activation function in the saturated or diverged region. Thereafter, with propagation processing, this phenomenon will be propagated in the entire model and will cause the corresponding gradient to vanish and explode.

This challenge can be overcome in several ways. For instance, we can use unsaturated activation such as Relu to relieve the problem to a certain degree. Moreover, the batch normalization [73] method, in which feature scaling is performed after convolution can be used; with this method, the result falls into the vanishing and exploding region can be avoided. In this experiment, we selected the simplest solution of adding depth to the most suitable degree, which can yield promising results while avoiding the gradient vanishing problem. Considering efficiency and accuracy, here, we added six convolutional layers into the original structure; as a result, we obtained testing accuracy and kappa value of 98.18% and 0.70 respectively.

#### 3.2.3. Influence of Window Size

The size of the input sample is a very significant factor that influences identification capability. Considering the image resolution and the characteristics of village buildings, the ideal window size must be slightly bigger than that for ordinary buildings, while information about a building’s surroundings must be included as well. The input window size of our original basic architecture is 18 × 18, which might be too small to extract enough valuable features.

Herein, we change the window size from 14 to 50 with intervals equal to 2; the parameter amount increases along with the increasing window size. For comparison, the experiment is conducted using a basic and a complex CNN structure, which is constructed based on the previous optimization principle. In particular, we focus on comparing the effect of window size on multiple relations, such as size 14 with 28, 16 and 32, etc., because a double-sized window contains the same information as a small one.

**Figure 4:**
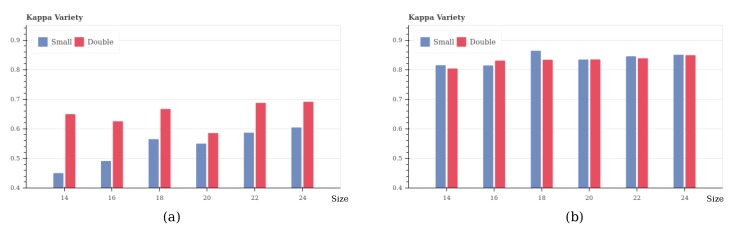
Window size in multiple relations. (**a**) with a simple structure; (**b**) with a complex structure.

From the testing result (Figure 4a), by implementing a simple structure, a double-sized window could yield better results, because it contains more abundant information that a small-sized window. However, if we implement a complex structure, although a double-sized window contains more information, we cannot always obtain better results (Figure 4b).

With the same CNN structure, bigger window size can obtain more features and parameters, but other methods such as adding filters and depth can also increase the amount of features. If the feature extraction capability of the model is weak, the big-size samples would help it to obtain more information than small-size ones, which would lead to good results. However, when we use a complex structure which can extract sufficient features, bigger window size can no longer yield good results, and extremely big window size might yield redundant and useless features, which lead to bad results. In this experiment, we choose a window size that is 50% bigger than in the ordinary architecture, with an adaptive number of kernels and depth. If the model suffers from over-fitting, herein, we also implement Dropout to address the problem.

In conclusion, to take full advantage of state-of-the-art CNN models, we optimized and enhanced them into new ones that match the village building identification task based on rigorous principles and experiments. Furthermore, we also visualized the representation of each layer to evaluate the feasibility of the model; here, take features extracted by VGGNet-like as an example. In later sections, we will introduce the self-designed models: AlexNet-like, VGGNet-like, GoogLeNet-like, and SqueezeNet-like.

### 3.3. AlexNet-Like

AlexNet is a revolutionary CNN architecture [27]. The parallel and merged structure of this architecture makes it suitable for extracting two sets of features while sharing information between the two sets. Deep CNN can be formulated elaborately with very high accuracy. Moreover, by running the model on GPUs implemented in CUDA, it becomes feasible to train the CNN model on large-scale datasets.

There are a few tricks of AlexNet in terms of both structure and processing. First, image preprocessing is conducted by only subsampling and feature scaling. Then, instead of the saturated activation method, AlexNet implements Relu, which is very efficient and six times faster than tanh [74], and it can avoid gradient vanishing and exploding to a certain degree. Third, given its parallel structure, AlexNet can be efficiently trained on multiple GPUs, and every GPU shares half kernels. To reduce over-fitting, AlexNet also employs tricks such as data augmentation, Dropout, and overlapping pooling structure. Finally, the stochastic gradient descent (SGD) method [75] is used with configurations such as weight decay, and gradually reducing momentum and learning rate.

**Figure 5:**
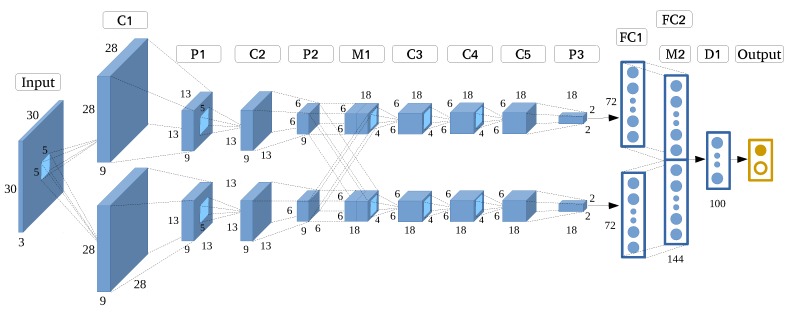
AlexNet-like architecture.

In this experiment, we rigorously optimized AlexNet into the AlexNet-like architecture as shown in Figure 5. To this end, we reduced the input size to 30 × 30, and optimized internal settings such as quantity of filter and kernel size based on the optimization principle, which increased the model’s efficiency by reducing the total number of parameters from about 60 million to 67,665.

### 3.4. VGGNet-Like

VGGNet is short for Very Deep Convolutional Networks. As its name suggests, VGGNet addresses the important aspect of CNN architecture design. The depth of this architecture makes it suitable for mining very deep and abstract features [28]. The architecture steadily increases the depth of networks by adding convolutional layers, and the quantity of filters gradually increases from the start to the end. Very small convolutional filters of size 3 × 3 are used in all layers, and the 1 × 1 filter can be seen as a linear transformation of the input channels. Other layers such as Zeroppading, Maxpooling, Flatten, Dense and Dropout also increase its identification capability. To avoid over-fitting, we must eliminate redundant features by using Dropout.

**Figure 6:**
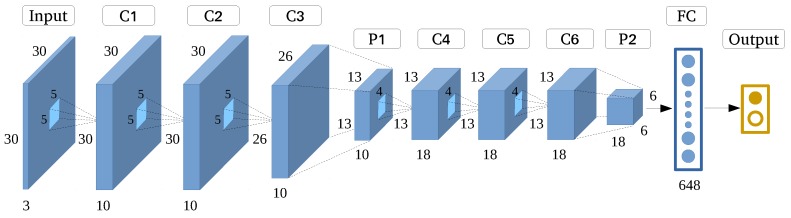
Very Deep Convolutional Network (VGGNet)-like architecture.

We propose the VGGNet-like architecture (Figure 6) in this experiment, which is very effective for identifying buildings in rural environments based on HRRS images. VGGNet-like is optimized by decreasing the depth quantity and filter size while retaining its original architecture. After optimization, the number of parameters decreases from 140 M to 70,453, which makes the model easy to train. The detailed settings are shown in Table 3.

**Figure 7:**
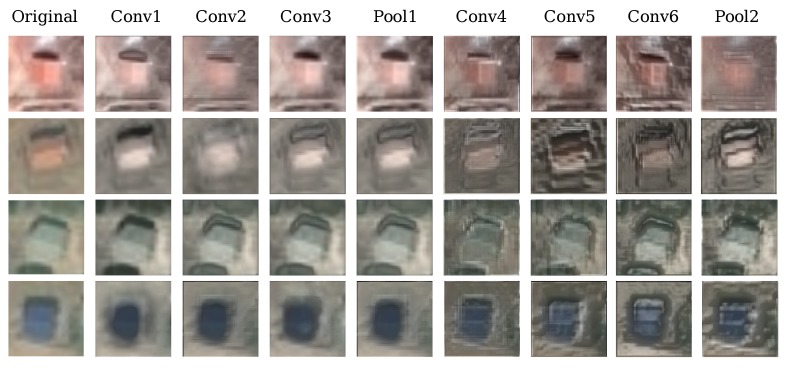
Reconstruction of Convolutional Neural Network (CNN) activations from different layers of VGGNet-like.

To intuitively understand the CNN activations for village buildings, we visualize the representations of each layer by reconstructing features from simple patterns to complex ones with the technique proposed in [76] using VGGNet-like, shown in Figure 7.

Due to the limitation of resolution, the external characteristics of village buildings cannot be shown clearly in some regions. However, the features extracted by convolutional layers still characterize village buildings well and can be reconstructed to images similar to the original image with more abstract information and blurriness as one progresses toward deeper layers. The visualization results also indicate the feature extraction capability of our self-designed models.

### 3.5. GoogLeNet-like

The main innovation of GoogleNet is its use of an architecture called Inception [29]. In general, Inception is a network in network structure, and the optimal local sparse structure of a vision network is spatially repeated from the start to the end. Three Inception structures used in different circumstances are introduced: typically, 1 × 1 convolution is used in Inception to compute reductions before the expensive 3 × 3 and 5 × 5 convolutions.

GoogleNet provides us with an inspiration of how to build a high-capability architecture. Most of the identification capability progress relies not only on more powerful hardware, large datasets and bigger models, but also and mainly on new ideas, algorithms, and improved network architectures.

**Figure 8:**
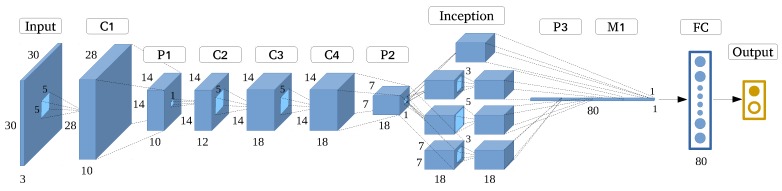
GoogleNet-like architecture.

By learning from GoogleNet, in this experiment, we built a GoogleNet-like structure as shown in Figure 8. We established the Inception architecture, while optimizing the number and sequence of layers and filters.

### 3.6. SqueezeNet-Like

Compared with other architectures, SqueezeNet has very few parameters while retaining similarly high accuracy [30]. It can achieve AlexNet-level accuracy with 50 times fewer parameters and <0.5 MB model size, in addition to identifying patterns by using very few parameters while preserving accuracy.

There are some tricks associated with its structure. First is the structure called fire, which appears like a fire blazing through a matchstick. Instead of the 3 × 3 convolutional core used in GoogLeNet, SqueezeNet uses 1 × 1 filters in a few layers, because 1 × 1 filters have one-ninth the number of parameters compared to 3 × 3 filters. The fire module comprises a squeeze convolution layer (consisting of only 1 × 1 filters), and the aforementioned layer is fed into an expanded layer comprising a mix of 1 × 1 and 3 × 3 convolutional filters. Then, the number of parameters can be decreased by decreasing the quantity of input channels. Third, downsampling is performed at a late stage in the network so that convolutional layers can have larger activation maps, which leads to higher classification accuracy. Finally, the output is directly generated by the pooling layer instead of the fully-connected layer, which can decrease the number of filters dramatically. For instance, the final convolutional layer obtains features of size 13 × 13 × 1000, and the pooling layer subsamples these features into size 1 × 1 × 1000, yielding 1000 possibilities in the process.

**Figure 9:**
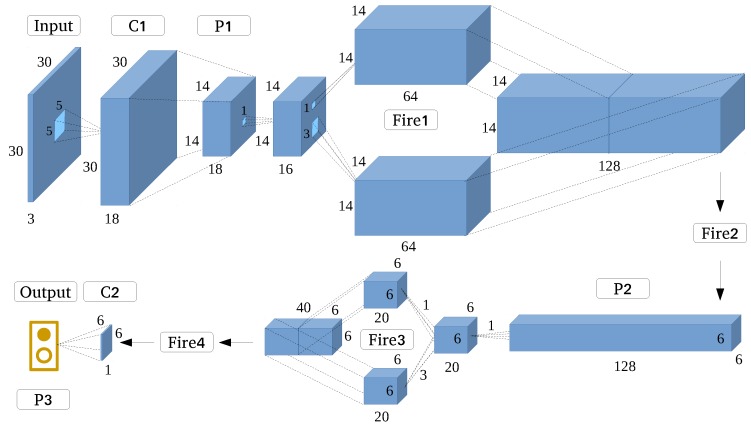
SqueezeNet-like architecture.

In this experiment, we designed a SqueezeNet-like architecture (Figure 9) starting from a standalone convolutional layer; then, we employed four fire modules. Emulating the original SqueezeNet structure, we gradually increased the number of filters per fire module from the start to end. Maxpooling (overlapping pooling) with stride was implemented after Conv1 and Merge2, and the final average pooling layer divides the output into two categories, namely, building and non-building.

### 3.7. Ensemble Convolutional Neural Networks

Very deep CNN structures with strong feature extraction capability are typically used for larger images measuring at least 200 × 200 pixels [31]. In the case of pixel-level village building identification, as analyzed in Section 3.2, small HRRS images are used to avoid redundant noise and information, while very deep structures and a large number of filters are not suitable owing to the problems of efficiency, accuracy, and robustness. Although the optimized state-of-the-art models can mine several features, a few important ones are inevitably lost. The feature extraction capability of an individual model is limited, and a promising solution is ensembling several CNN models into a stronger model by using the multiscale feature learning method.

**Figure 10:**
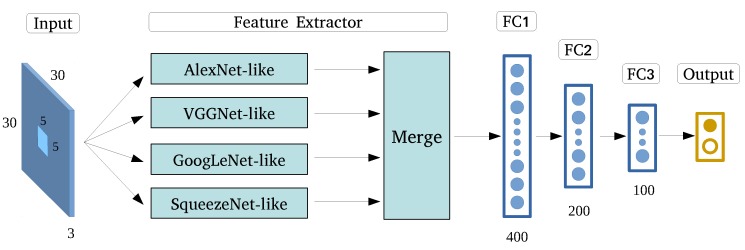
Ensemble Convolutional Neural Networks.

Here, we present ECNN, shown in Figure 10, an elaborate CNN frame formulated based on the ensembling of optimized state-of-the-art CNN models, followed by three layers of neural networks and softmax to implement classification. Instead of varying the contextual input size, multiscale feature learning can be achieved by inputting HRRS images of the same size to all CNNs. This would also help preserve integrated building information.

By taking full advantage of the different optimized state-of-the-art models’ feature extraction capabilities, the proposed ECNN structure can achieve better classification results. Moreover, it can solve the problem of remaining small input image size, while avoiding the serious problems caused by very deep CNNs, such as gradient vanishing. To the best of our knowledge, there is no existing related CNN structure to identify village buildings by using HRRS images, and the feasibility of ECNN will be evaluated in the following sections.

## 4. Result and Discussion

We defined the CNN model utilized in our previous study [24] as basic CNN structure, and it cannot achieve a stable, high kappa value in many testing areas. Moreover, the building identification capability of the corresponding model is relatively limited. As shown in the previous chapter, based on the rigorous CNN model optimization and construction principle, we formulated four types of self-designed structure by using state-of-the-art networks and ensembled them into the ECNN model.

In this chapter, to compare and discuss the village building identification capability of different models, we first employ the same dataset and study area used in [24]. Thereafter, we use the models to implement village building identification in practice. We discuss and evaluate the feasibility of the model in terms of kappa coefficient, overall accuracy, confusion matrix, standard deviation, and computation efficiency.

### 4.1. Comparison of Different Models

[-20]Here, we set the experimental parameters as follows: number of iterations = 300, window size = 30 × 30, learning rate = 0.03, activation Relu, and Softmax. In terms of dataset, 50,655 and 12,664 images were selected as the training and cross validation samples respectively. Because the land feature information of non-building areas is much more abundant than building areas in villages, 13,319 are positive samples and 50,000 are negative samples. For the sake of comparison, we selected the same testing area as in our previous study in Laos. The number of filters and depth information were different for each architecture. The employed parameter details and the training results are listed in Table 4.

The training results show that all proposed self-designed CNN models outperformed the basic ones and achieved very high accuracy of over 99% with much higher efficiency. Thereafter, we implemented the trained models for testing, and the results in terms of overall accuracy, kappa value, and confusion matrix are given in Table 5.

From the testing results, the self-designed models performed much better than the basic structure, and the accuracy and kappa coefficient increased by about 2.5% and 0.3, respectively. The confusion matrix shows that TP and TN increased substantially, while FP and FN decreased, which means misclassification in the cases of building and non-building areas was solved to a certain degree. In particular, ECNN, which can achieve a kappa coefficient of up to 0.85, outperformed other methods. The testing results indicate the feasibility of the model optimization method and the strong capability of the proposed ECNN method, which is based on ensembling the feature extraction parts of the state-of-the-art models for village building identification.

### 4.2. Implementation of CCNs

In this section, we present the building identification results obtained in the study areas in Laos and Kenya. These results were obtained using the optimized state-of-the-art CNN models and ECNN. In addition, we discuss their feasibility in terms of accuracy, stability, and efficiency.

To evaluate and compare the robustness of different CNN models, here, we deliberately selected several representative and typical small-segment areas, where land features and buildings present different characteristics in terms of color, external structure, and texture. The concrete numerical results are presented in terms of kappa coefficient, standard deviation, and mean average overall accuracy, while the intuitive classification results are presented in terms of different colors, where green refers to true positive, that is, the actual buildings are classified correctly as buildings; blue indicates the non-building areas that were incorrectly labeled as buildings; red indicates the buildings that were marked incorrectly as non-buildings; and black indicates true negative, which denotes the correctly classified non-building areas.

**Figure 11:**
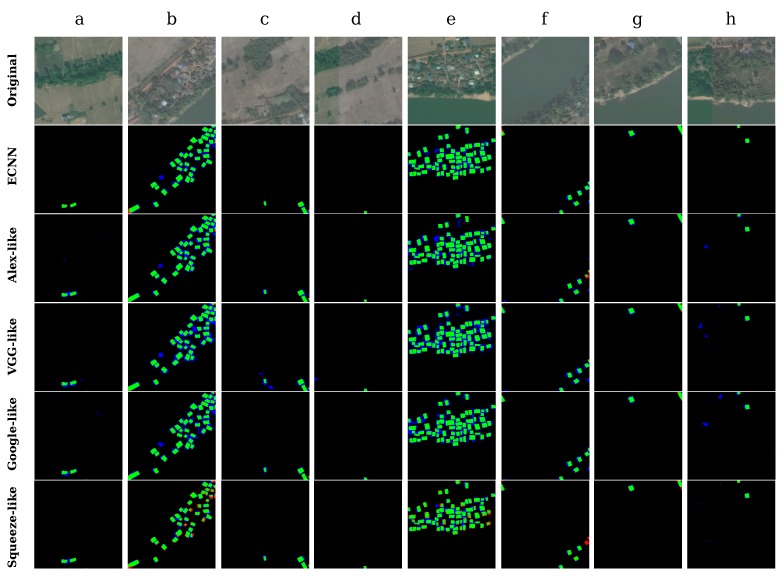
Identification results of eight small segments in bank regions.

Because villages along river banks are representative of the landscape in many countries [77], we selected a few related regions, as shown in the top row of Figure 11. Notably, the regular outline of the bank in some regions is quite similar to buildings, which makes identification very challenging in many cases. The testing result obtained using the proposed different CNNs (Figure 11, second row to the final row) shows the models’ excellent identification capability in such regions, and the majority of buildings are correctly identified, while other land features such as river bank are also well classified. However, in Figure 11c,f,h, some regions with vegetation cover are misclassified as buildings, and buildings near the boundary are marked as non-building areas by the optimized state-of-the art models, while ECNN correctly classified these regions and identified buildings with higher accuracy. In Figure 11b, there is a region where non-building areas are misclassified by ECNN; after carefully analyzing the original image, we believe that this was caused by imperfect ground truth.

As shown in Table 6, the proposed ECNN model outperformed the other models, achieving an average kappa of 0.82 and overall accuracy of 98.34% in regions (a–h). In terms of standard deviation, ECNN is slightly better than a few other models, but the kappa can be relatively unstable when the density of buildings is high in a given region.

**Figure 12:**
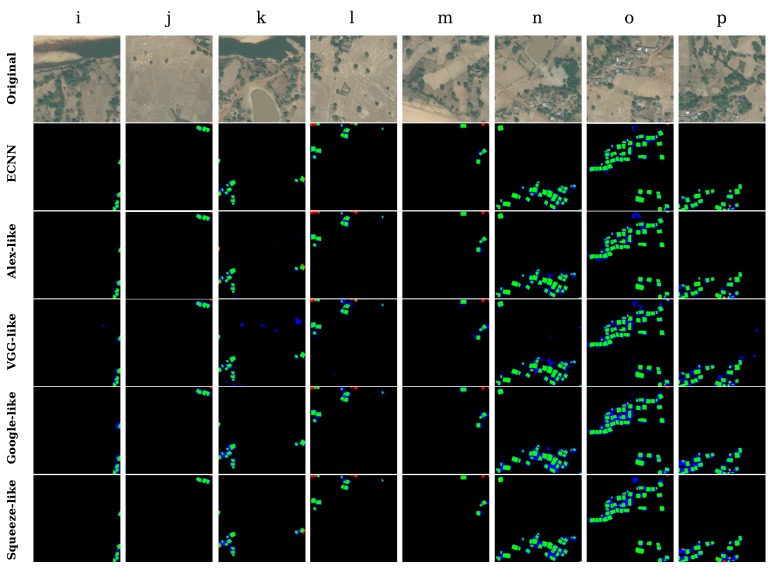
Identification results of eight small segments in mixed-type regions.

The complex and mixed-type village regions that contain an abundance of terrestrial features such as streams, pools, vacancies, vegetation, and crops were selected for conducting the comparison. As shown in Figure 12, ECNN could identify buildings in all cases, and it yielded the least false positive results compared to the other models.

The detailed results are given in Table 7. ECNN not only achieved the highest average kappa of 0.77 and overall accuracy of 98.13%, but also the best standard deviation of 0.02. By contrast, the individual optimized state-of-the-art models yield unstable performance with average kappa values ranging from 0.67 to 0.74. This indicates that the proposed ECNN offers higher robustness and better feasibility within complex testing regions compared to individual CNN models.

**Figure 13:**
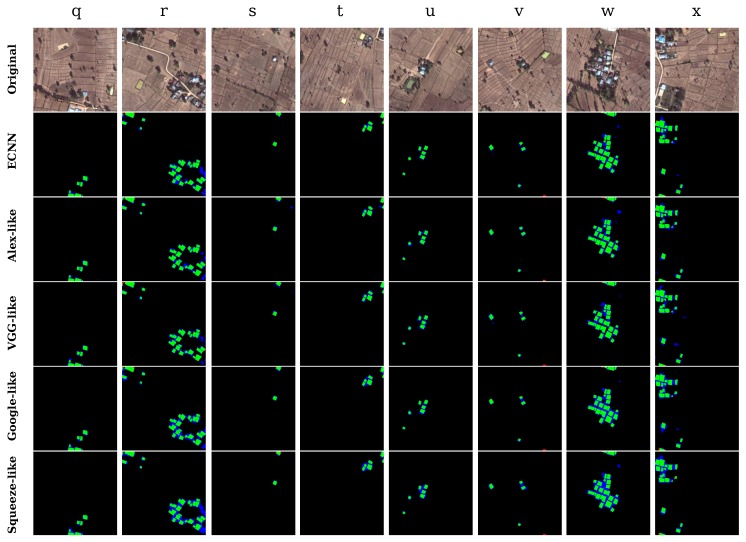
Identification results of eight small segments in artificial land regions.

As shown in Figure 13, finally, we selected typical areas containing plenty of human-built land features such as roads, agricultural fields, and pounds. Owing to the similar textures and external structures to the buildings, artificial land features are prone to misclassification, leading to decreased accuracy of the results along with a large number of false positives. According to the testing results in Figure 13 (second row to the final row), although ECNN can achieve better performance than the other models, a few artificial land features such as roads and yards are inevitably identified as buildings. It indicates that the ECNN model still needs to be enhanced by training it using a more diverse training dataset.

The results in Table 8 infer that in regions with complex artificial land features, ECNN can achieve a very high kappa of 0.80 and overall accuracy of 98.38%, while the SqueezeNet-like model can achieve a kappa of only 0.72.

It should be noted that a comparison of the models’ feasibility in all cases and other regions that are not included in these study areas is very difficult because they differ in terms of resolution, data acquisition methods, reference datasets, and class definitions. However, from the testing results, it can be concluded that the optimized state-of-the-art models, especially ECNN, can achieve comparably efficient village building identification results to the previous best result in the tested study areas. Moreover, the proposed ECNN model has considerably better accuracy and robustness than the individual optimized CNN model structure in the village building identification task.

## 5. Conclusions

In this study, we proposed a novel CNN frame called ECNN for village building identification using HRRS images. First, we constructed four self-designed CNN structures based on state-of-the-art CNN models and a rigorous optimization principle. Then, to extract most of their identification capabilities, we ensembled the feature extractor parts of each individual optimized model and concatenated them into ECNN based on the multiscale feature learning method. Finally, the generated ECNN was applied to a pixel-level village building identification task in developing countries.

The experimental results show the potential and the capability of the proposed ECNN model and the optimized state-of-the-art models in village building identification. The models achieved considerably higher accuracy than the previous best methods. In particular, the proposed ECNN model achieved considerably higher accuracy, and the kappa value improved from the previous best of 0.57 to 0.86 and overall accuracy from 96.64% to 99.26%. It outperformed the individual optimized CNN models as well, which indicates the feasibility of our proposed method.

More detailed exploration of the method is required in the future. First, to test the robustness of the method, regions of different resolution, as well as various data acquisition methods and reference datasets, need to be tested. Second, multi-class village landscape classification needs to be implemented using the proposed method. Finally, in case there are any limitations in the training data source, transfer learning [78] and the generative model [79] will be applied to enhance the proposed ECNN model.

## Figures and Tables

**Table 1:** Relationship between number of filters and accuracy.

		Training				Testing		
Structure	Para	Acc (%)	Kappa	Epoch (s)	Total (min)	Acc (%)	Kappa	Total (s)
Ori	1669	95.31	0.86	1.41	7.06	97.29	0.60	1.59
×3	11917	98.94	0.97	1.57	7.85	98.15	0.72	1.93
×9	97957	99.19	0.98	2.29	11.46	98.22	0.73	3.31
×25	0.73M	99.73	0.99	7.09	35.44	98.98	0.83	10.16
×100	11.57M	99.69	0.99	83.09	415.46	98.79	0.80	88.00
×200	46.18M	97.54	0.92	299.06	1459.27	98.14	0.70	5.67

**Table 2:** Relationship between depth and accuracy.

		Training				Testing		
Structure	Para	Acc (%)	Kappa	Epoch (s)	Total (min)	Acc (%)	Kappa	Total (s)
Ori	1669	93.02	0.79	1.49	74.29	94.58	0.42	1.73
+2 Conv	4891	96.30	0.89	2.06	103.16	96.84	0.58	2.60
+4 Conv	8133	96.21	0.88	2.73	136.93	98.06	0.68	3.55
+6 Conv	11,335	97.45	0.92	3.44	172.09	98.18	0.70	4.65
+8 Conv	14,557	97.54	0.92	4.16	208.19	98.14	0.70	5.67
+10 Conv	17,779	97.80	0.93	4.94	247.03	97.67	0.65	6.73
+12 Conv	21,001	78.97	0.00	6.18	308.87	97.53	0.00	7.81

**Table 3:** VGGNet-like architecture

Layer	Output Shape	Kernel Size	Scale	Para	Connect to
Input	(30, 30, 3)	-	-	-	-
Conv 1	(30, 30, 10)	(5, 5)	-	760	Input
Conv 2	(30, 30, 10)	(5, 5)	-	2510	Conv 1
Conv 3	(26, 26, 10)	(5, 5)	-	2510	Conv 2
Pooling 1	(13, 13, 10)	-	2,2	0	Conv 3
Conv 4	(13, 13, 18)	(4, 4)	-	2898	Pooling 1
Conv 5	(13, 13, 18)	(4, 4)	-	5202	Conv 4
Conv 6	(10, 10, 18)	(4, 4)	-	5202	Conv 5
Pooling 2	(5, 5, 18)	-	2, 2	0	Conv 6
Flatten	(648)	-	-	0	Pooling 2
Output	(1)	-	-	649	Flatten
Total Parameters: 19,731					

**Table 4:** Training result by different CNNs.

	Parameter		Training		
Structure	Original	New	Acc (%)	Kappa	Epoch (s)
ECNN	-	506,288	99.78	0.99	31.21
AlexNet-like	60.97 M	51,249	99.77	0.99	5.42
VGGNet-like	143.67 M	70,453	99.78	0.99	13.81
GoogLeNet-like	7.00 M	37,589	99.71	0.99	6.62
SqueezeNet-like	1.25 M	39,941	99.73	0.99	7.23
Basic	-	4349	96.48	0.90	98.22

**Table 5:** Testing result by different CNNs.

	Testing			Confusion Matrix			
Structure	Acc	Kappa	Total (s)	TN	FP	FN	TP
ECNN	99.15	0.85	56.22	522,162	4519	56	13,263
AlexNet-like	98.95	0.82	16.72	521,048	5633	37	13,282
VGGNet-like	98.95	0.82	25.77	52,1058	5623	50	13,269
GoogLeNet-like	98.91	0.81	12.19	520,837	5844	63	13,256
SqueezeNet-like	98.89	0.81	17.93	520,713	5968	45	13,274
Basic	96.64	0.57	180.70	509,295	17,366	799	12,540

**Table 6:** Testing results in bank regions with different CNNs.

Structure	a	b	c	d	e	f	g	h	Mean	Std	Acc_Mean (%)
ECNN	**0.86**	**0.76**	**0.83**	**0.91**	**0.76**	**0.78**	**0.86**	**0.84**	**0.82**	0.05	**98.34**
AlexNet-like	0.72	0.74	0.80	0.79	0.73	0.73	0.82	0.69	0.75	0.04	98.06
VGGNet-like	0.74	0.73	0.82	0.76	0.73	0.77	0.81	0.53	0.74	0.08	98.00
GoogLeNet-like	0.80	**0.76**	**0.83**	0.80	**0.76**	0.76	0.84	0.77	0.79	**0.03**	98.30
SqueezeNet-like	0.69	0.68	0.65	0.63	0.68	0.70	0.82	0.61	0.68	0.06	97.49

**Table 7:** Testing results in mixed-type regions with different CNNs.

Structure	i	j	k	l	m	n	o	p	Mean	Std	Acc_Mean(%)
ECNN	**0.79**	**0.81**	**0.75**	**0.73**	**0.76**	**0.76**	**0.77**	**0.78**	**0.77**	**0.02**	**98.13**
AlexNet-like	**0.79**	0.79	0.72	0.69	0.74	0.75	0.74	0.75	0.74	0.03	98.00
VGGNet-like	0.70	0.72	0.62	0.67	0.64	0.70	0.71	0.67	0.68	0.03	97.25
GoogLeNet-like	0.63	0.74	0.68	0.67	0.67	0.66	0.68	0.65	0.67	0.03	97.63
SqueezeNet-like	0.74	0.79	0.65	0.70	0.72	0.71	0.70	0.66	0.71	0.04	97.50

**Table 8:** Testing results of different CNNs in artificial land regions.

Structure	q	r	s	t	u	v	w	x	mean	std	Acc_mean
ECNN	**0.84**	**0.78**	**0.87**	**0.84**	**0.81**	**0.76**	**0.78**	**0.79**	**0.80**	0.04	**98.38**
AlexNet-like	0.82	0.70	0.81	0.75	0.75	0.75	0.73	0.72	0.75	0.04	98.25
VGGNet-like	0.81	0.78	0.75	0.78	0.72	0.75	0.76	0.77	0.77	**0.03**	98.32
GoogLeNet-like	0.81	0.74	0.77	0.78	0.73	0.73	0.77	0.75	0.76	**0.03**	98.04
SqueezeNet-like	0.77	0.65	0.81	0.72	0.69	0.68	0.70	0.70	0.72	0.05	98.30

## Abbreviations

The following abbreviations are used in this manuscript:

HRRS	High Resolution Remote Sensing
GE	Google Earth
CNN	convolutional neural networks
ECNN	ensemble convolutional neural networks
SGD	Stochastic Gradient Descent
GIS	Geographic Information System
SAR	synthetic aperture radar
ILSVRC	ImageNet Large Scale Visual Recognition Challenge
AlexNet	CNN architecture developed by Alex Krizhevsky
VGGNet	very deep CNN architecture developed by Simonyan
GoogLeNet	CNN architecture developed by Christian Szegedy
SqueezeNet	CNN architecture developed by Forrest
RNN	recurrent neural networks
